# Dehydroandrographolide attenuates Toll-like receptor signaling by dual inhibition of MyD88- and TRIF-dependent pathways

**DOI:** 10.1038/s41598-026-47514-6

**Published:** 2026-04-16

**Authors:** Ye Eun Lee, Hanbin Ko, Dongwoo Lee, Ha Eun Park, Seo-Yeong Kim, Gyuri Park, Seonah Kim, Younghyun Lee, Hyung-Sun Youn, Gyo Jeong Gu

**Affiliations:** 1 Department of Medical Science, Graduate School, SoonChunHyang University, Asan-si, Chungnam 31538 Republic of Korea; 2 Department of Biomedical Laboratory Science, College of Medical Sciences, SoonChunHyang University, Asan-si, Chungnam 31538 Republic of Korea

**Keywords:** Anti-inflammation, Dehydroandrographolide, MyD88-dependent pathway, Toll-like receptor signaling, TRIF-dependent pathway, Cell biology, Diseases, Drug discovery, Immunology, Molecular biology

## Abstract

Toll-like receptors (TLRs) are key mediators of innate immune responses, and their dysregulation contributes to inflammatory diseases. Dehydroandrographolide (DAG), a diterpene lactone from *Andrographis paniculata*, is known for its anti-inflammatory activity, but its effects on individual branches of TLR signaling remain unclear. RAW264.7 and 293T cells were used to evaluate the effects of DAG on MyD88- and TRIF-dependent signaling pathways. Luciferase reporter assays, Western blotting, RT-PCR, and nitrite assays were employed to assess NF-κB and IRF3 activation and inflammatory mediator expression. Statistical analysis was performed using one-way ANOVA. DAG significantly inhibited activation of NF-κB and IRF3 induced by TLR agonists (LPS, MALP-2, and Poly[I:C]) and by overexpression of MyD88- and TRIF-associated downstream molecules. Correspondingly, DAG suppressed iNOS, IFNβ, and IP-10 expression, indicating dual inhibition of both TLR signaling branches. DAG exerts dual inhibitory effects on MyD88- and TRIF-dependent TLR signaling, attenuating inflammatory mediator expression. DAG may represent a promising molecular scaffold or mechanistic candidate for modulating TLR-mediated inflammatory signaling.

**Supplementary Information:**

The online version contains supplementary material available at 10.1038/s41598-026-47514-6.

## Introduction

Inflammation represents one of the body’s primary defense responses against microbial invasion and tissue injury^1^. This process involves intricate interactions among various cell types and signaling molecules, typically accompanied by vasodilation, increased leukocyte recruitment, and the release of pro-inflammatory mediators^2^. Such physiological changes play a crucial role in safeguarding damaged tissue and initiating its repair^3^. Based on duration and nature, inflammation can be classified into acute and chronic forms. Acute inflammation develops quickly in response to injury, infection, or tissue damage and is generally short-lived^4^. In contrast, chronic inflammation persists over extended periods, often resulting from continuous irritation or unresolved injury, and it can cause structural tissue alterations and damage. Persistent inflammatory activity is implicated in the onset and progression of many inflammatory disorders^5^.

Toll-like receptors (TLRs) are a well-characterized subgroup of pattern recognition receptors (PRRs) that detect conserved molecular patterns from microbes and trigger immune responses^6^. They mediate the inflammatory reaction against pathogens including bacteria, viruses, and fungi, serving as a vital bridge between the innate and adaptive branches of immunity. TLRs also sense pathogen-associated molecular patterns (PAMPs) and damage-associated molecular patterns (DAMPs) to initiate and fine-tune inflammatory cascades^7^. Ten types of TLRs have been identified in humans and thirteen in mice; they are located either on the plasma membrane or within endosomal compartments^8^. Membrane-localized TLRs generally recognize structural components of microbial surfaces, such as lipoproteins, lipopolysaccharides, and flagellins, whereas endosomal TLRs specialize in detecting microbial nucleic acids like double-stranded RNA, single-stranded RNA, or unmethylated CpG DNA^9^. Upon ligand recognition, TLRs transmit signals through two main routes: the MyD88- and the TRIF-dependent pathway. The MyD88-dependent route, used by most TLRs, primarily promotes the expression of inflammatory cytokines and chemokines. Meanwhile, the TRIF-dependent pathway is engaged by certain TLRs and induces production of interferons, while also regulating NF-κB via interaction with RIP1^10^.

Dysregulation of TLR signaling has been linked to a variety of inflammatory and autoimmune disorders, as well as infectious diseases^11^. Thus, agents capable of modulating TLR pathways represent promising tools for controlling inflammation. Notably, medicinal plants—many of which possess both anti-cancer and anti-inflammatory activities—offer a valuable source of bioactive compounds that may support immune regulation and inflammation management^12^.

**Fig. 1:**
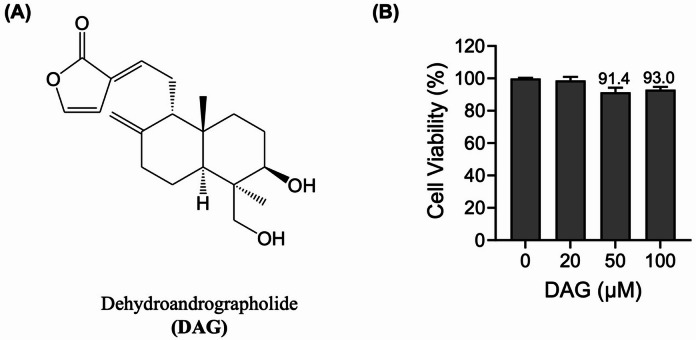
The structure of dehydroandrographolide (DAG) (**A**) and Cell viability assay (**B**). RAW264.7 cells were treated with epoxomicin (20, 50, or 100 μM) for 4 h. The CellTiter 96 AQ_ueous_ One Solution Reagent (20 μl/well) was added directly to culture wells. The plate was incubated at 37 °C for 4 h in a humidified, 5% CO_2_ atmosphere. The absorbance was recorded at 490 nm with a 96-well plate reader. Results show representative results of 3 independent experiments. Values are expressed as the mean ± SEM.

*Andrographis paniculata*, belonging to the family *Acanthaceae*, has been traditionally utilized across China, India, and numerous Southeast Asian regions for centuries, primarily owing to its notable antipyretic and anti-inflammatory activities^13^. In traditional Chinese medicine, *Andrographis paniculata* has been prescribed for the management of persistent or recurrent infectious and inflammatory disorders, particularly those affecting the upper respiratory tract and causing intestinal diarrhea^14–16^. Among its bioactive constituents, dehydroandrographolide (DAG) has been identified as a compound with substantial immunomodulatory potential (Fig. 1A). Despite these findings, the precise molecular mechanisms by which DAG regulates TLR-mediated immune responses remain incompletely understood.

Previous studies have reported that DAG suppresses NF-κB activation downstream of TLR4 stimulation, primarily within the MyD88-dependent signaling axis^17^. In addition, the anti-inflammatory effects of DAG have been demonstrated in Poly(I:C)-induced inflammatory models, indicating its efficacy against viral RNA–mimetic stimuli^18^. However, whether DAG also modulates TRIF-dependent TLR signaling pathways, which play a critical role in IRF3 activation and type I interferon responses, has not been systematically investigated. In the present study, we examined the effects of DAG on both MyD88- and TRIF-dependent branches of TLR signaling using multiple TLR agonists and downstream signaling components. By dissecting these distinct signaling cascades, we aimed to determine whether DAG functions as a dual-pathway regulator of TLR-mediated inflammatory responses.

## Methods

### Aim, design, and setting of the study

The aim of this study was to investigate the anti-inflammatory mechanisms of dehydroandrographolide (DAG), focusing on its regulatory effects on Toll-like receptor (TLR) signaling pathways. Specifically, we sought to determine whether DAG modulates both MyD88- and TRIF-dependent signaling cascades, which are two major branches of TLR-mediated immune responses.

This study was designed as an in vitro experimental study using murine macrophage RAW264.7 cells and human embryonic kidney 293 T cells, which are well-established models for analyzing TLR signaling and transcriptional activation. The experiments were conducted under controlled laboratory conditions at the Department of Biomedical Laboratory Science, Soonchunhyang University (Asan, Republic of Korea).

### Reagents

DAG was purchased from Cayman chemical and diluted in dimethylsulfoxide(DMSO). Lipopolysaccharide (LPS) was obtained from List Biological Laboratories (Ann Arbor, Michigan, USA; Cat. No.36841). Macrophage-activating lipopeptide-2 (MALP-2) was purchased from Alexis Biochemical (San Jose, CA, USA; Cat.No.421). Polyinosinic-polycytidylic acid (Poly[I:C]) was purchased from InvivoGen (San Diego, CA, USA; Cat.No. tlrl-pic-5). All other reagents were purchased from Sigma-Aldrich (St. Louis, MO, USA) unless otherwise described.

### Cell culture

RAW264.7 cells (a murine monocytic cell line; ATCC TIB-71) and human embryonic kidney 293 T cells (ATCC CRL-3216) were cultured in Dulbecco’s modified Eagle’s medium(DMEM) supplemented with 10% (v/v) fetal bovine serum, 100 units/ml penicillin, and 100 μg/ml streptomycin at 37 °C with 5% CO_2_ atmosphere in a humidified incubator until confluence.

### Cell viability test

Cell viability was assessed using a 3-(4,5-dimethylthiazol-2-yl)-5(3-carboxymethoxyphenyl)-2-(4-sulfophenyl)-2H-tetrazolium (MTS)-based colorimetric assay. RAW 264.7 cells were treated with each compound for 4 h. Twenty microliters of the CellTiter 96 AQueous One Solution reagent (Promega, Madison, WI, USA) was added directly to the culture wells. The plate was then incubated for 4 h in a humidified, 5% CO_2_ atmosphere, after which the absorbance at 490 nm was recorded using a 96-well plate reader.

### Transfection and luciferase reporter gene assay

Cells were seeded into 48-well plates at a density of 0.8 × 10^5^ cells/ml and incubated at 37 °C in a 5% CO_2_/95% air environment. RAW264.7 and 293 T cells were transfected with a luciferase plasmid and a HSP70‐β‐galactosidase plasmid as an internal control using a p3000 and lipofectamine (Invitrogen, Carlsbad, CA, USA) according to the manufacturer’s instruction. Cells were then treated with LPS, MALP-2, and Poly[I:C] for 8 h after being treated with DAG (20, 50 μM) for 1 h. The 8 h stimulation period was selected to allow sufficient accumulation of downstream effector proteins, such as iNOS, while avoiding potential secondary or compensatory effects associated with prolonged stimulation. Luciferase and β‐galactosidase enzyme activities were determined using a Luciferase Assay System (Promega). Luciferase activity was normalized against β‐galactosidase activity.

### Western blotting analysis

Cells were seeded into 6‐well plates at a density of 1.0 × 10^6^ cells/ml and incubated at 37 °C in a 5% CO_2_/95% air environment for 48 h. RAW264.7 cells were pretreated with DAG (20, 50 μM) for 1 h. They were then treated with agonists for 8 h. Total protein was extracted from cell lysates using RIPA lysis buffer. Cell lysates were subjected to 12% and 14% sodium dodecyl sulfate–polyacrylamide gel electrophoresis to separate proteins. Proteins were then transferred to polyvinylidene difluoride (PVDF) membranes. Membranes were blocked with PBS containing 0.1% Tween 20 and 5% nonfat dry milk for 24 h to prevent non-specific binding of antibodies. After the blocking step, membranes were incubated with specific primary antibodies and secondary antibodies conjugated with horseradish peroxidase (GE Healthcare, Chicago, IL, USA). Reactive bands were visualized using a Western Blot Detection System (iNtRON, Songnam, Korea). Membranes were stripped with 0.5 N NaOH for 20 min to reprobe with different antibodies.

### Nitrite assay

RAW 264.7 macrophages at a concentration of 0.8 × 10^5^ cells/ml were seeded in 48-well plates and incubated for 24 h. Subsequently, they were treated with each compound in the presence or absence of LPS, MALP-2, or Poly[I:C] for 18 h. Samples (100 μl) of the culture medium were incubated with 150 μl Griess reagent (1% sulfanilamide and 0.1% naphthylethylene diamine in a 2.5% phosphoric acid solution) at room temperature for 5 min in a 96-well microplate. The absorbance at 570 nm was read using a plate reader, and the concentration of NO was then determined by the preparation of a standard calibration curve, using sodium nitrite as the standard.

### Real-time RT-PCR analysis of IFNβ expression

Total RNA was extracted using Ribospin™ (GeneAll, Seoul, Korea) according to the manufacturer’s instructions. Total RNA (5 µg) was reverse-transcribed using a HyperScript™ for RT-PCR (GeneAll) and amplified with a Step One Plus Real-Time PCR System (Applied Biosystems) using a Power SYBR Green PCR Master kit (Applied Biosystems). Primers used to detect mouse IFNβ were as follows: forward primer 5′-TCCAAGAAAGGACGAACATTCG-3′, and reverse primer 5′-TGAGGACATCTCCCACGTCAA-3′. Primers for mouse β-actin (used as an internal control) were: forward primer 5′-TCATGAAGTGTGACGTTGACATCCGT-3′, and reverse primer 5′-CCTAGAAGCATTTGCGGTGCACGATG-3′. The following PCR conditions were used: denaturation at 95 °C for 10 min; and 40 cycles of denaturation at 95 °C for 15 s, annealing at 56 °C for 30 s, and extension at 72 °C for 30 s. The specificity of PCR was assessed using a melting curve analysis. Fold induction of IFNβ expression was measured by real-time PCR in triplicate experiments relative to the vehicle control.

### In vitro kinase assay for IKKβ and TBK1

In vitro kinase activities of IKKβ and TBK1 were measured using the IKKβ Kinase Enzyme System (Promega) and the TBK1 Kinase Enzyme System (Promega), respectively, according to the manufacturer’s instructions. Briefly, recombinant active IKKβ or TBK1 was incubated with the provided kinase substrate peptide and ATP in kinase reaction buffer in the presence or absence of DAG (20 or 50 μM). Kinase reactions were carried out at room temperature for 1 h. The reactions were terminated by addition of the supplied detection reagent, and kinase activity was quantified by measuring luminescence using a microplate reader. Relative kinase activity was calculated by normalizing luminescence values to the vehicle-treated control, which was set to 1.0. All experiments were performed in triplicate.

### Data analysis

Data were obtained from triplicate experiments. Values were expressed as the mean ± standard error of the mean (SEM). Differences in the data were evaluated using one-way analysis of variance (ANOVA), followed by Tukey’s multiple comparisons test. A P-value of less than 0.05 was considered statistically significant. All analyses were conducted using the GraphPad Prism software version 10.3 (GraphPad Software, San Diego, CA, USA).

## Results

### Cytotoxicity of DAG

The cytotoxic effect of DAG was evaluated in RAW 264.7 cells using a 3-(4,5-dimethylthiazol-2-yl)-5-(3-carboxymethoxyphenyl)-2-(4-sulfophenyl)-2H-tetrazolium (MTS)-based colorimetric assay. Cell viability was 91.4% at 50 μM DAG and decreased to 93.0% at 100 μM DAG (Fig. 1B). To minimize potential non-specific effects at higher concentrations, 50 μM DAG was selected as the maximum concentration for most subsequent mechanistic experiments.

### DAG suppresses NF-κB activation induced by TLR4 or TLR2 and TLR6 agonists

**Fig. 2:**
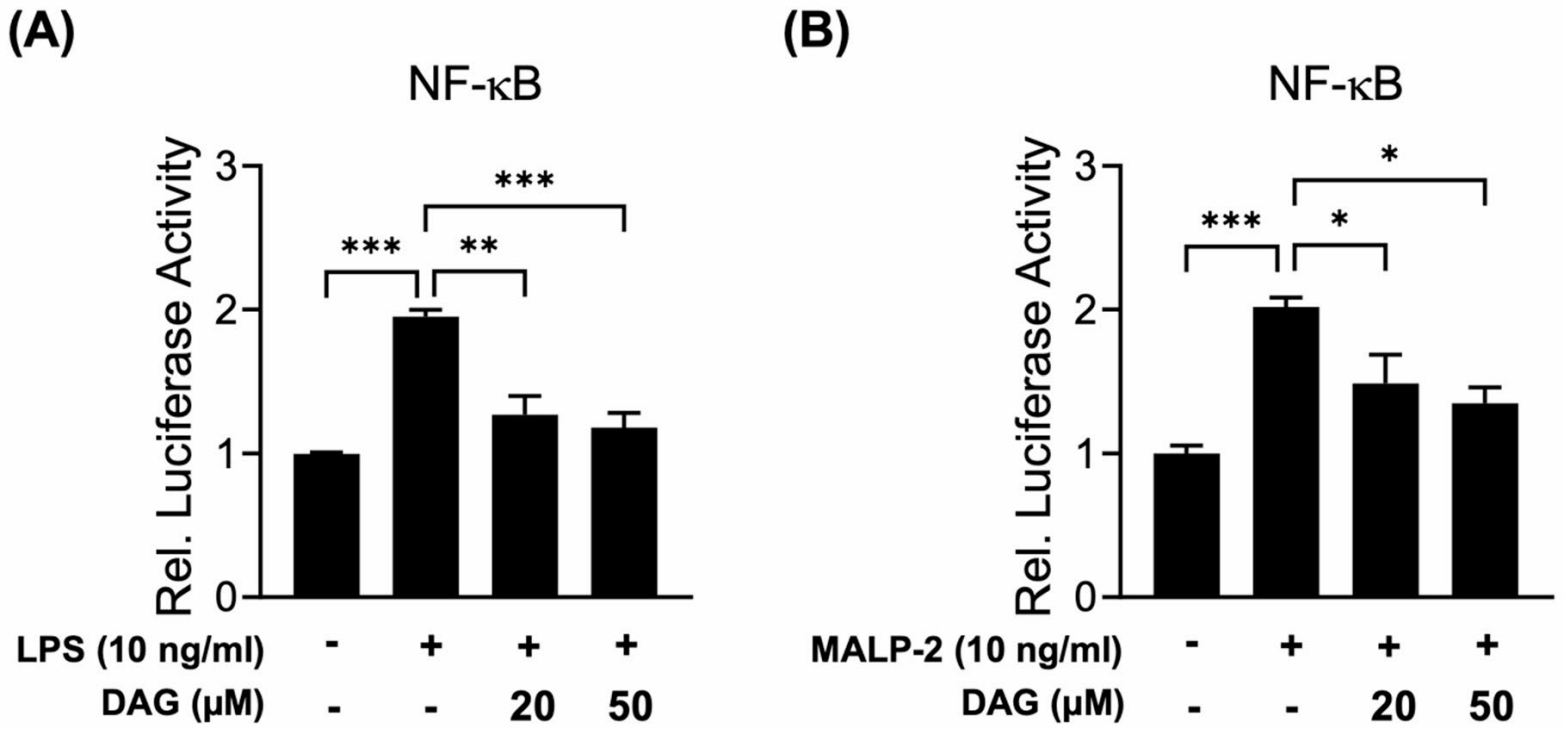
DAG suppresses NF-κB activation induced by LPS and MALP-2. (**A**, **B**) RAW264.7 cells were transfected with NF-κB luciferase reporter plasmid, pre-treated with DAG (20 or 50 μM) for 1 h, and then treated with LPS or MALP-2 for an additional 8 h. Cell lysates were prepared and the luciferase enzyme activities were determined. Relative luciferase activity was normalized with β-gal activity. Results show representative results of 3 independent experiments. Values are expressed as the mean ± SEM. Statistical significance was determined using a one-way ANOVA. *p < 0.05; **p < 0.01; ***p < 0.001.

NF-κB is a transcription factor that plays an important role in the human immune response^19^, and activation of NF-κB through the MyD88-dependent pathways and TRIF-dependent pathways leads to inflammation by regulating the expression of several target genes. This study, therefore, examined whether LPS (TLR 4 agonist) or MALP-2 (TLR 2 and TLR 6 agonist) inhibits the activity of NF-κB using a luciferase reporter gene assay. The results indicate that DAG significantly inhibited NF-κB induced by LPS (Fig. 2A) or MALP-2 (Fig. 2B). This result indicates that DAG can inhibit the activity of NF-κB by modulating the signal transduction system through TLRs.

### DAG suppresses iNOS expression induced by TLR4 or TLR2 and TLR6 agonists

**Fig. 3:**
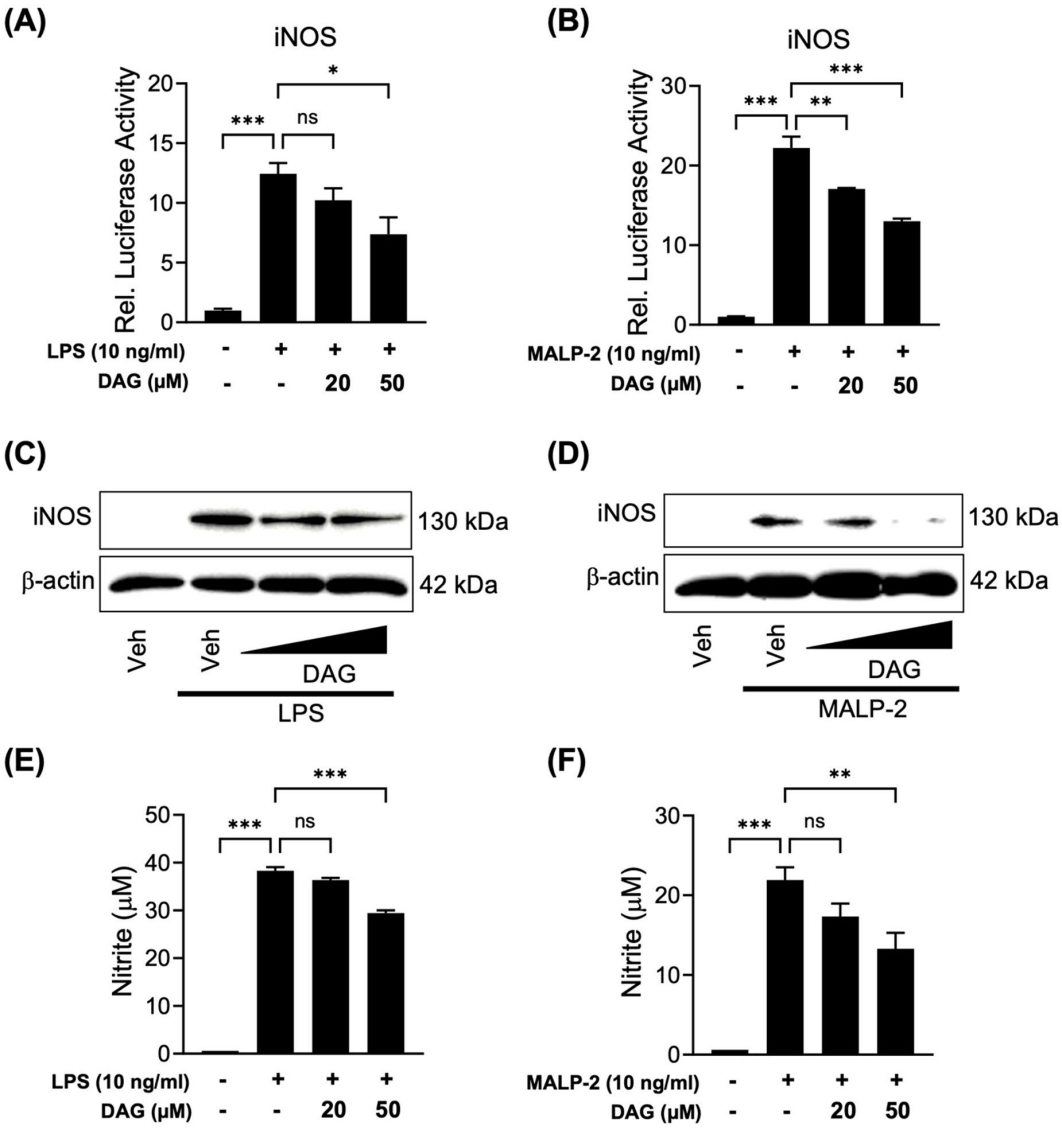
DAG inhibits iNOS expression induced by LPS and MALP-2. (**A**, **B**) RAW264.7 cells were transfected with iNOS luciferase reporter plasmid and pretreated with 20 or 50 μM DAG for 1 h and then treated with LPS (**A**) or MALP-2 (**B**) for an additional 8 h. Cell lysates were prepared and luciferase enzyme activities were determined. Relative luciferase activity was normalized with β-gal activity. (**C**, **D**) RAW264.7 cells were pretreated with 20 or 50 μM DAG for 1 h and then further stimulated with LPS (**C**) or MALP-2 (10 ng/ml) (**D**) for an additional 8 h. Cell lysates were analyzed for iNOS and β-actin protein by immunoblots. (**E**, **F**) RAW 264.7 cells were pretreated with 20 or 50 μM DAG for 1 h and then treated with LPS (**E**), or MALP-2 (**F**), for an additional 20 h. The amounts of nitrite in supernatant were measured using Griess reagent. Results show representative results of 3 independent experiments. Values are expressed as the mean ± SEM. Statistical significance was determined using a one-way ANOVA. *p < 0.05; **p < 0.01; ***p < 0.001.

When TLRs recognize various agonists and send signals downstream, they induce the activation of NF-κB. Activated NF-κB induces the expression of inflammatory genes such as iNOS^20^. Therefore, this study investigated whether DAG could modulate iNOS expression induced by LPS or MALP-2. According to the iNOS-luciferase reporter gene assay (Fig. 3A, B) and Western blot analysis (Fig. 3C, D), DAG inhibited iNOS expression induced by LPS or MALP-2 in RAW 264.7 cells. Additionally, we were confirmed that the concentration of nitrite produced by iNOS decreased (Fig. 3E, F).

### DAG suppresses NF-κB activation induced by MyD88 downstream signaling components of TLRs

**Fig. 4:**
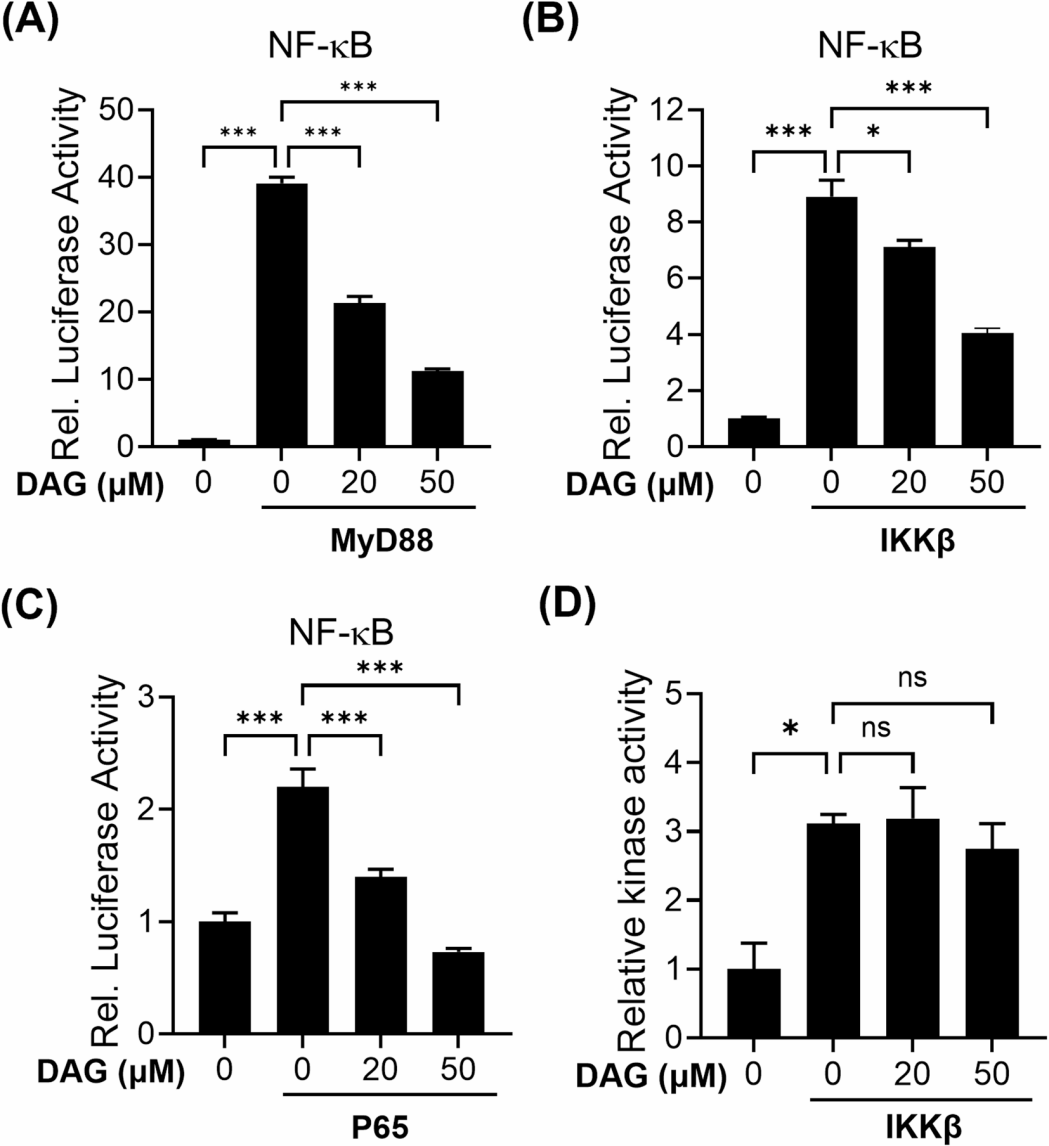
DAG suppresses NF-κB activation induced by downstream signaling components of MyD88-dependent pathway. (A-C) 293 T cells were co-transfected with NF-κB luciferase reporter plasmid and the expression plasmid of MyD88 (**A**), IKKβ (**B**), or p65 (**C**). Cells were further treated with DAG (20 or 50 μM) for 18 h. Relative luciferase activity was normalized with β-gal activity. Results show representative results of 3 independent experiments. (**D**) In vitro kinase activity of IKKβ was measured in the presence of DAG (20 or 50 μM). Relative kinase activity is shown compared with the untreated control. Data represent one of three independent experiments with similar results and are expressed as the mean ± SEM. Statistical significance was determined by one-way ANOVA. *p < 0.05; ***p < 0.001; ns, Not significant.

MyD88-dependent pathway is the canonical adaptor of the inflammatory signaling pathway of Toll-like receptors^21^. From the above results, we found that DAG could inhibit the activity of NF-κB through the TLR signaling pathway. Therefore, we conducted experiments to determine whether DAG could also regulate the activation of NF-κB through the MyD88 signaling pathway. We transfected MyD88, IKKβ, and p65, which are downstream components of MyD88 dependent pathway, into 293 T cells and performed a luciferase reporter gene assay. The results indicate that NF-κB induced by MyD88 (Fig. 4A), IKKβ (Fig. 4B), and p65 (Fig. 4C) is significantly inhibited by DAG, indicating that DAG modulates signaling events downstream of MyD88 within the NF-κB activation cascade.

To further examine whether DAG directly inhibits key catalytic components of TLR signaling, in vitro kinase assays were performed for IKKβ. DAG did not significantly affect the kinase activity of IKKβ under the experimental conditions tested (Fig. 4D). These results indicate that DAG does not act as a direct catalytic inhibitor of the principal kinases downstream of the MyD88-dependent pathways.

### DAG suppresses IRF3 activation induced by TLR4 agonist

**Fig. 5:**
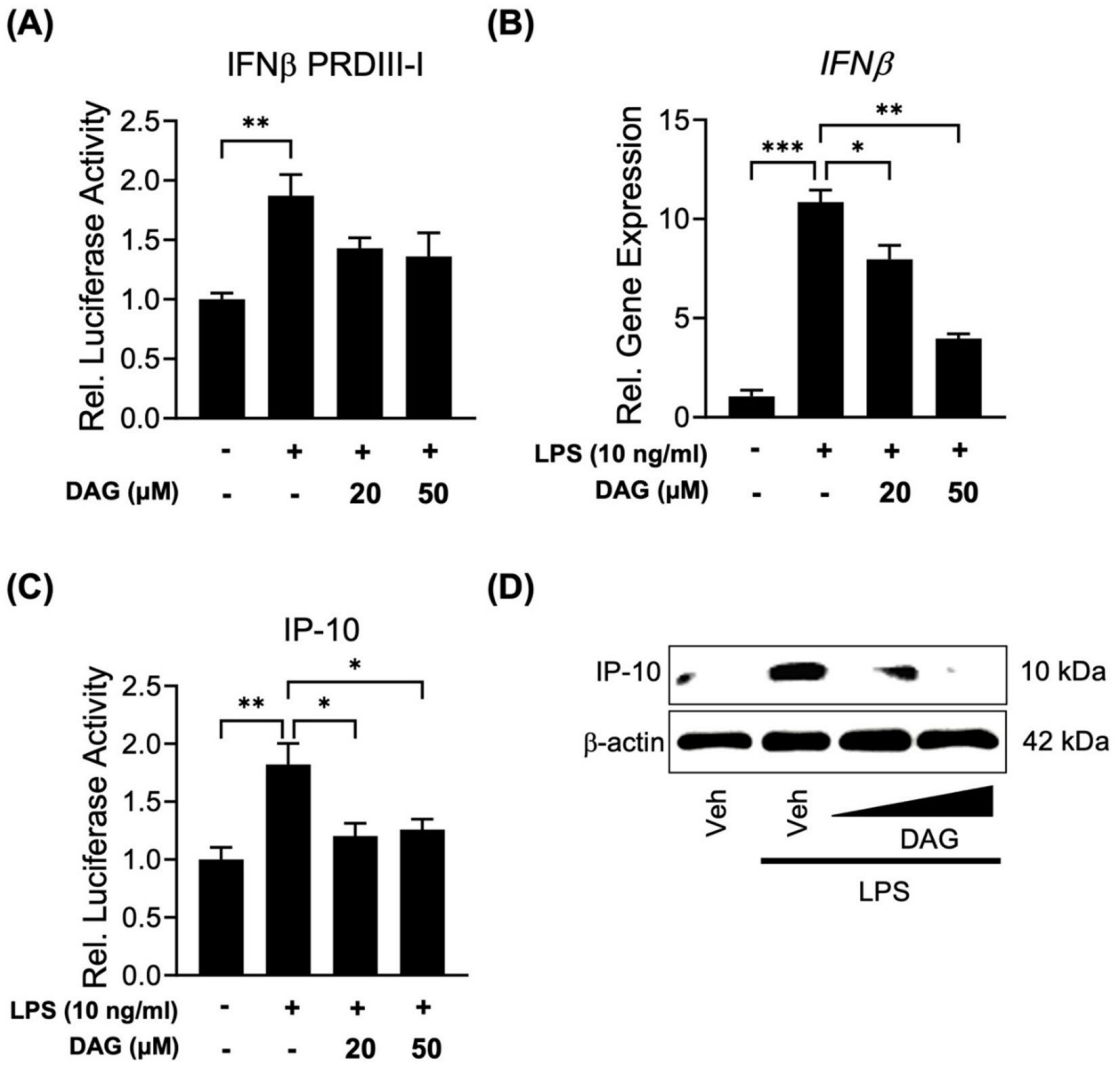
Epoxomicin suppresses IRF3 activation induced by LPS. (**A**) RAW264.7 cells were transfected with IRF3 binding site (IFNβ PRDIII-I) luciferase reporter plasmid, pre-treated with DAG (20 or 50 μM) for 1 h, and then treated with LPS for an additional 8 h. Cell lysates were prepared and the luciferase and β-galactosidase enzyme activities were measured. luciferase activity was normalized with β-gal activity. (**B**) RAW264.7 cells were treated with DAG (20 or 50 μM) for 1 h and further stimulated with LPS for 18 h. Total RNAs were extracted and the levels of IFNβ expression were determined by quantitative real-time RT-PCR analysis. IFNβ expression was normalized with β-actin (internal control) expression. (**C**) RAW 264.7 cells were transfected with IP-10-luciferase reporter plasmid, pre-treated with DAG (20 or 50 μM) for 1 h, and then treated with LPS for an additional 8 h. Cell lysates were prepared and the luciferase and β-galactosidase enzyme activities were measured. luciferase activity was normalized with β-gal activity. (**D**) RAW 264.7 cells were pre-treated with DAG (20 or 50 μM) for 1 h and then treated with LPS for a further 8 h. Cell lysates were analyzed for IP-10 and β-actin protein by immunoblots. Veh, Vehicle; DAG, Dehydroandrographolide.

IRF3 is activated exclusively through the TRIF signaling pathway. Therefore, we used LPS (TLR4 agonist) to activate TLR4, which signals through both the MyD88-dependent pathway and TRIF-dependent pathways. Therefore, it is employed to determine whether DAG can regulate the TRIF signaling pathway. IFN-β was selected as a representative transcriptional readout of TRIF–IRF3–dependent signaling, and fold induction was used to quantitatively assess pathway modulation by DAG. Using a luciferase reporter gene assay with the IFNβ promoter domain containing the IRF3 binding site (IFNβ PRDIII-I), we confirmed that DAG significantly inhibited the activity of IRF3 induced by LPS (Fig. 5A). These results are further confirmed by RT-PCR (Fig. 5B). To further investigate the regulation of TRIF by DAG, we examined the expression of IP-10, so we confirmed by IP-10-luciferase reporter gene assay (Fig. 5C) and Western blot (Fig. 5D) that IP-10 induced by LPS is significantly inhibited by DAG.

### DAG suppresses NF-κB activation and iNOS expression induced by TLR3 agonist

**Fig. 6:**
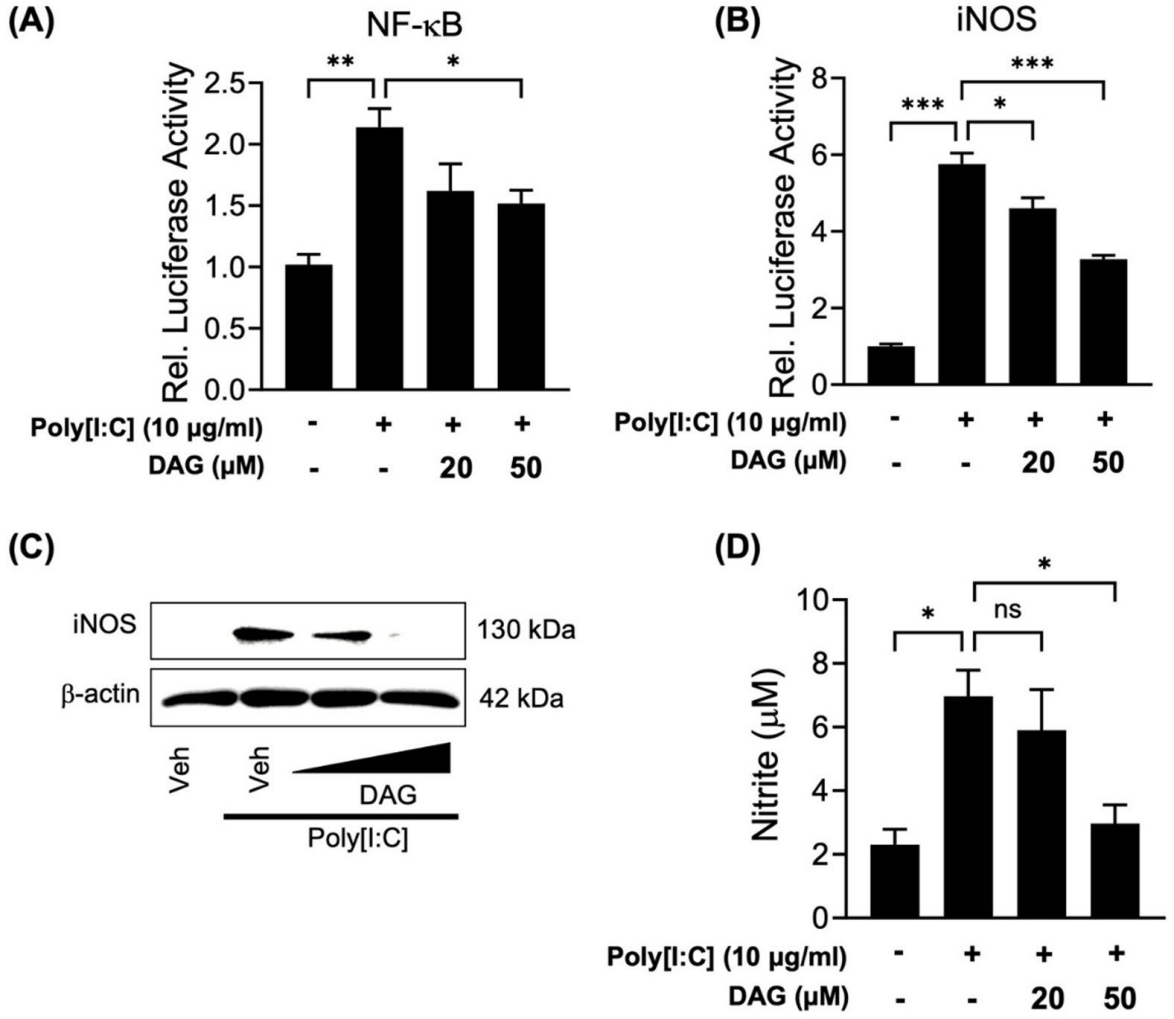
DAG suppresses NF-κB activation induced by Poly[I:C]. (**A**) RAW264.7 cells were transfected with NF-κB luciferase reporter plasmid, pre-treated with DAG (20 or 50 μM) for 1 h, and then treated with Poly[I:C] for an additional 8 h. Cell lysates were prepared and the luciferase enzyme activities were determined. (**B**) RAW264.7 cells were transfected with iNOS luciferase reporter plasmid and pretreated with DAG (20 or 50 μM) for 1 h and then treated with Poly[I:C] for an additional 8 h. Cell lysates were prepared and luciferase enzyme activities were determined. (**C**) RAW264.7 cells were pretreated with 20 or 50 μM DAG for 1 h and then further stimulated with Poly[I:C] for an additional 8 h. Cell lysates were analyzed for iNOS and β-actin protein by immunoblots. (**D**) RAW 264.7 cells were pretreated with 20 or 50 μM DAG for 1 h and then treated with Poly[I:C] for an additional 20 h. The amounts of nitrite in the supernatant were measured using Griess reagent. Results show representative results of 3 independent experiments. Values are expressed as the mean ± SEM. Statistical significance was determined using a one-way ANOVA. *p < 0.05; **p < 0.01; ***p < 0.001.

TLR3 exclusively utilizes the TRIF pathway. Therefore, we used Poly[I:C] (a TLR3 agonist) to observe the regulation of NF-κB via the TRIF pathway. Our results showed that NF-κB induced by Poly[I:C] (Fig. 6A) is significantly inhibited by DAG. This finding indicates that DAG can inhibit NF-kB activation through the TRIF-dependent pathway. Additionally, we confirmed that DAG inhibits Poly[I:C] induced iNOS expression using the iNOS-luciferase reporter gene assay (Fig. 6B) and Western blot analysis (Fig. 6C). Furthermore, a reduction in nitrite levels is observed in the nitrite assay (Fig. 6D).

### DAG suppresses IRF3 activation induced by TLR3 agonist

**Fig. 7:**
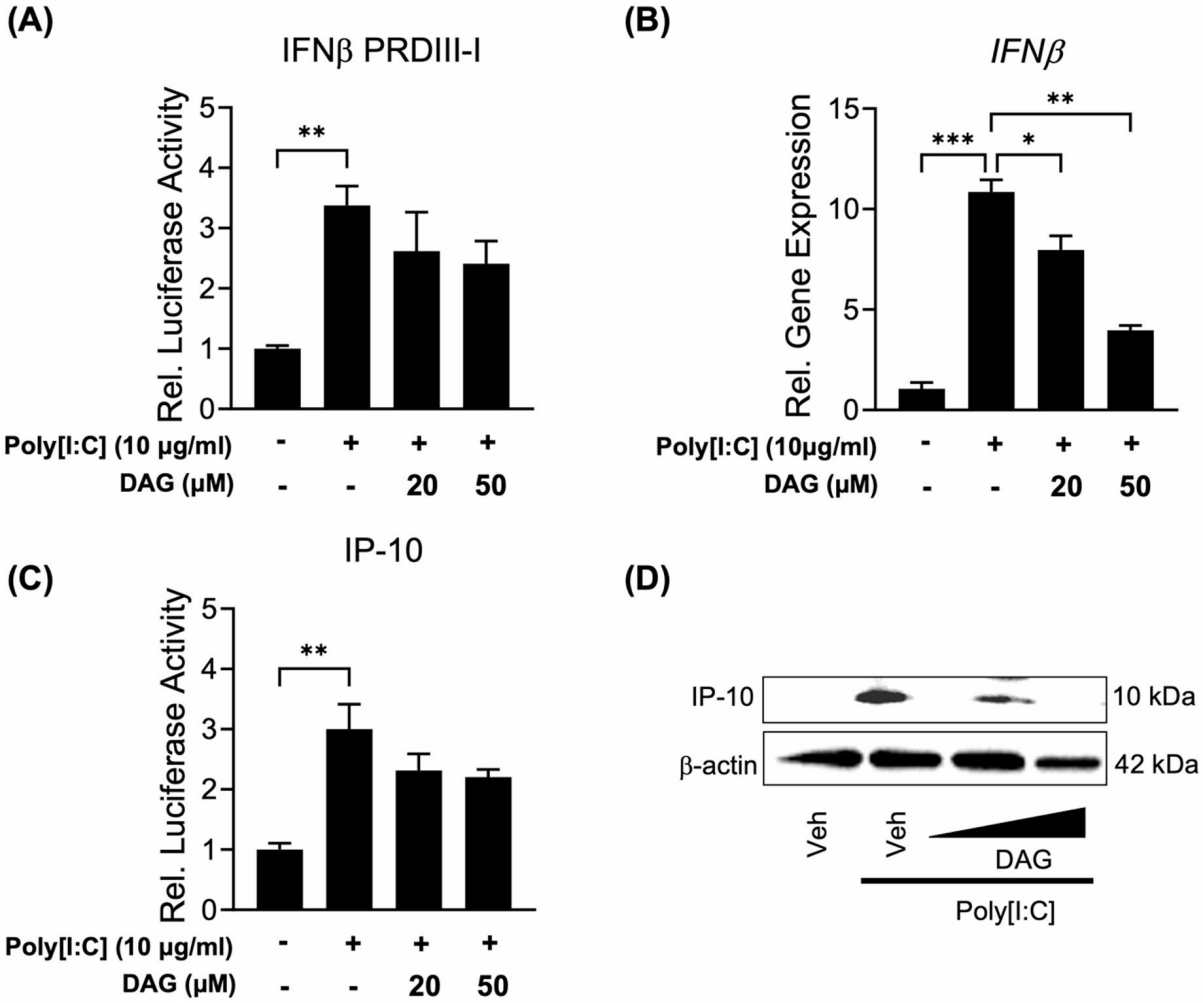
DAG suppresses IRF3 activation induced by Poly[I:C]. (**A**) RAW264.7 cells were transfected with IRF3 binding site (IFNβ PRDIII-I) luciferase reporter plasmid, pre-treated with DAG (20 or 50 μM) for 1 h, and then treated with Poly[I:C] for an additional 8 h. Cell lysates were prepared and the luciferase and β-galactosidase enzyme activities were measured. (**B**) RAW264.7 cells were treated with DAG (20 or 50 μM) for 1 h and further stimulated with Poly[I:C] for 18 h. Total RNAs were extracted and the levels of IFNβ expression were determined by quantitative real-time RT-PCR analysis. IFNβ expression was normalized with β-actin (internal control) expression. (**C**) RAW 264.7 cells were transfected with IP-10-luciferase reporter plasmid, pre-treated with DAG (20 or 50 μM) for 1 h, and then treated with Poly[I:C] for an additional 8 h. Cell lysates were prepared and the luciferase and β-galactosidase enzyme activities were measured. (**D**) RAW 264.7 cells were pre-treated with DAG (20 or 50 μM) for 1 h and then treated with Poly[I:C] for a further 8 h. Cell lysates were analyzed for IP-10 and β-actin protein by immunoblots. Results show representative results of 3 independent experiments. Values are expressed as the mean ± SEM. Statistical significance was determined using a one-way ANOVA. *p < 0.05; **p < 0.01; ***p < 0.001. Veh, Vehicle; DAG, Dehydroandrographolide.

**Fig. 8:**
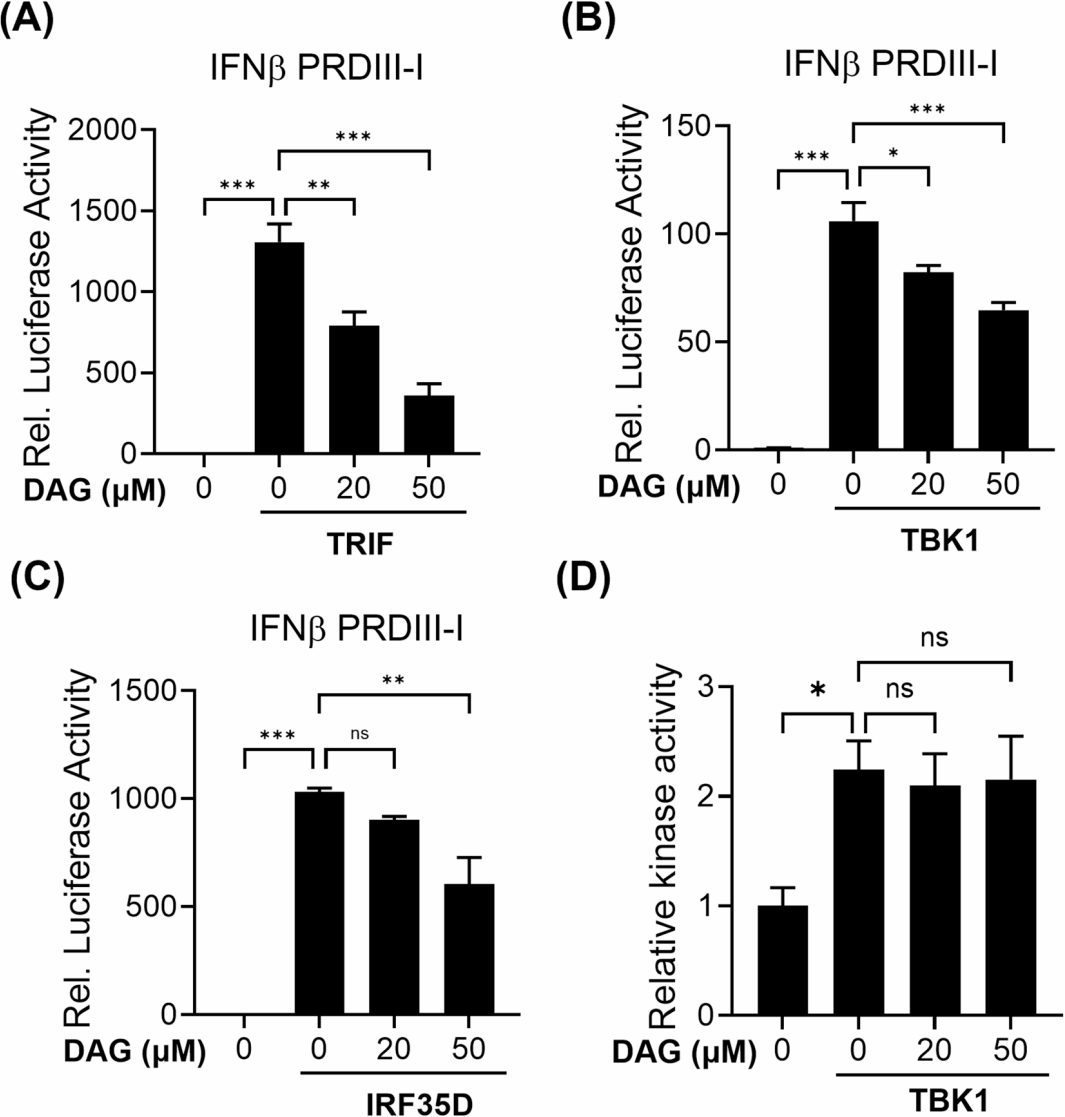
DAG suppresses IRF3 activation induced by downstream signaling components of TRIF-dependent pathway. (**A**–**C**) 293 T cells were co-transfected with IRF3 binding site (IFNβ PRDIII-I)-luciferase reporter plasmid and the expression plasmid of TRIF (**A**), TBK1 (**B**), or IRF3 (**C**). Cells were further treated with DAG (20 or 50 μM) for 18 h. luciferase activity was normalized with β-gal activity. (**D**) In vitro kinase activity of TBK1 was measured in the presence of DAG (20 or 50 μM). Relative kinase activity is shown compared with the untreated control. Results show representative results of 3 independent experiments. Values are expressed as the mean ± SEM. Statistical significance was determined using a one-way ANOVA. *p < 0.05; **p < 0.01; ***p < 0.001.

We investigated whether DAG could inhibit the activation of IRF3 induced by the TLR3 agonist Poly[I:C]. The results showed that DAG significantly inhibited Poly[I:C] induced IRF3 activation, as shown by the luciferase reporter gene assay (Fig. 7A). This inhibition is further confirmed by RT-PCR analysis (Fig. 7B). Additionally, the IP-10 luciferase reporter gene assay (Fig. 7C) and Western blot analysis (Fig. 7D) also corroborated that DAG can inhibit IRF3 activation. The results indicate that DAG can regulate the TRIF signaling system. Therefore, we conducted experiments to identify molecular targets within the TRIF signaling system. TRIF, TBK1, and IRF3 are TRIF downstream components and are transfected into 293 T cells to perform luciferase reporter gene assay. These findings indicate that IRF3 activation induced by TRIF (Fig. 8A), TBK1 (Fig. 8B), and IRF3-5D (Fig. 8C) is significantly inhibited by DAG, suggesting that DAG broadly suppresses TRIF-dependent signaling pathways leading to IRF3 activation.

To further examine whether DAG directly inhibits key catalytic components of TRIF-dependent TLR signaling, in vitro kinase assays were performed for TBK1. DAG did not significantly affect the kinase activity of TBK1 under the experimental conditions tested (Fig. 8D). These results indicate that DAG does not act as a direct catalytic inhibitor of TBK1 in the TRIF-dependent pathway.

**Fig. 9:**
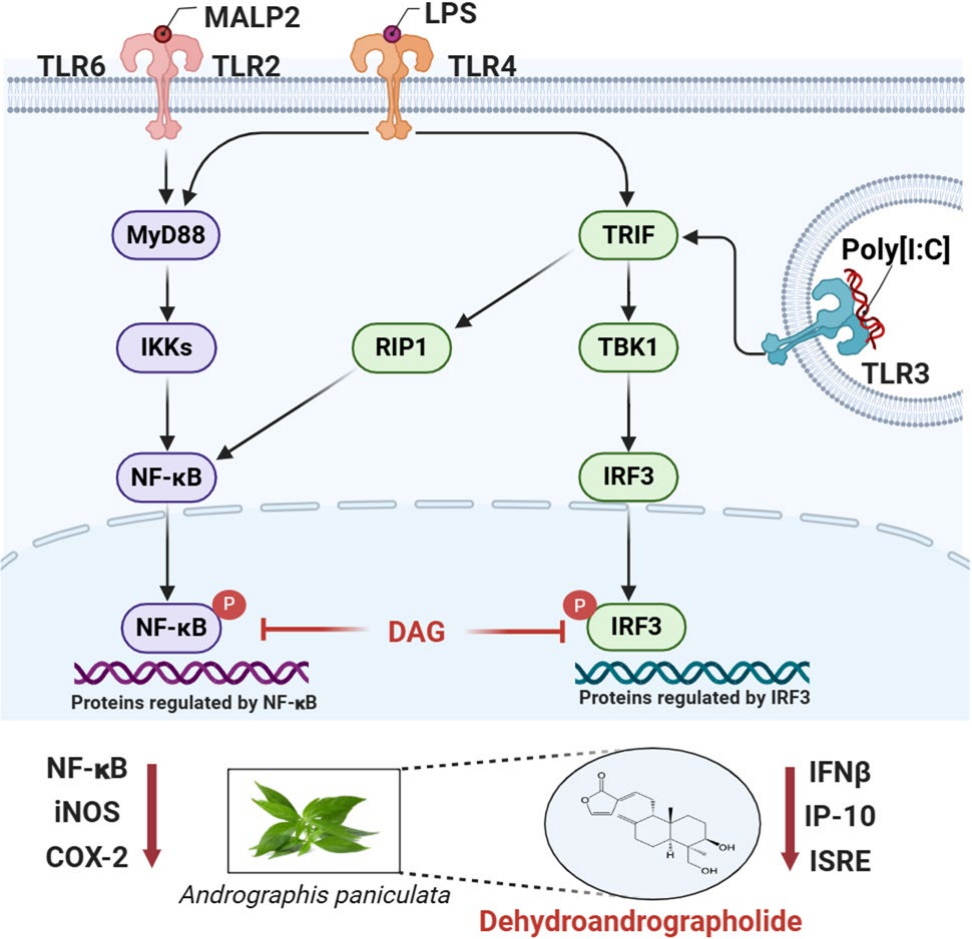
Dehydroandrographolide (DAG) suppresses Toll-like receptor (TLR) signaling by inhibiting both MyD88- and TRIF-dependent pathways. This dual blockade attenuates downstream activation of NF-κB and IRF3, leading to reduced expression of pro-inflammatory mediators such as iNOS, COX-2, IFNβ, and IP-10. These findings highlight DAG as a potential anti-inflammatory agent derived from *Andrographis paniculata*.

## Discussion

This study demonstrates that DAG exerts broad inhibitory effects on Toll-like receptor (TLR)–mediated inflammatory responses by targeting both MyD88-dependent and TRIF-dependent pathways^22^. Using three distinct TLR agonists, LPS, MALP-2, and Poly[I:C], we observed that DAG attenuated downstream activation of key transcription factors, including NF-κB and IRF3, and consequently reduced the expression of inflammatory mediators such as iNOS and IP-10. These findings highlight a multifaceted role of DAG in modulating innate immune signaling across distinct TLR pathways. LPS, a well-characterized ligand for TLR4, activates both MyD88- and TRIF-mediated cascades, resulting in robust induction of proinflammatory cytokines. MALP-2, in contrast, signals primarily through TLR2 and TLR6, engaging the MyD88-dependent route. Poly[I:C], as a synthetic analog of viral double-stranded RNA, activates TLR3 within endosomes and preferentially signals through TRIF^23^.

In this study, we show that DAG suppresses IRF3 activation and interferon-related gene expression downstream of both TLR4 and TLR3 stimulation. Moreover, our adaptor- and kinase-level analyses indicate that DAG inhibits signaling events downstream of TRIF, including TBK1- and IRF3-mediated transcriptional activation. These findings suggest that the regulatory effects of DAG are not confined to a single adaptor pathway or receptor context, but instead span distinct branches of the TLR signaling network. Thus, the novelty of this work lies in the identification of DAG as a dual-pathway modulator of TLR signaling, rather than in a simple extension of TLR agonist usage.

A notable aspect of this study is the concurrent suppression of NF-κB and IRF3 activation. NF-κB is a central regulator of inflammatory gene expression, including iNOS, which plays a critical role in nitric oxide production and inflammation resolution^24^. IRF3, on the other hand, is essential for antiviral defense, driving type I interferon production and chemokine expression such as IP-10^25,26^. The simultaneous downregulation of these two transcription factors indicates that DAG’s action is not restricted to a single branch of the TLR signaling network but instead encompasses broader regulatory control. This observation expands upon previous findings that primarily focused on the TLR4–NF-κB axis, suggesting that DAG may influence common signaling intermediates shared by multiple TLRs rather than acting at the receptor level alone^17,27^.

From a therapeutic perspective, these results suggest that DAG could serve as a promising candidate for treating diseases characterized by excessive or chronic inflammation. By targeting both MyD88- and TRIF-dependent pathways, DAG may offer an advantage over agents that selectively inhibit a single signaling route.

Furthermore, the suppression of both proinflammatory and antiviral signaling suggests potential applicability in conditions where immune overactivation contributes to pathology, such as autoimmune disorders or viral-induced hyperinflammation. Notably, this dual regulatory property aligns with the traditional use of *Andrographis paniculata* extracts for managing systemic inflammatory and infectious conditions, thereby providing a molecular rationale that bridges empirical herbal efficacy with modern immunological mechanisms^27^.

Importantly, our in vitro kinase assays revealed that DAG did not measurably inhibit the catalytic activity of either IKKβ or TBK1 under the experimental conditions tested. This finding suggests that the observed suppression of NF-κB- and IRF3-dependent transcriptional responses is unlikely to be mediated through direct inhibition of these key kinases. Instead, DAG may modulate TLR signaling through non-catalytic regulatory mechanisms, such as interference with adaptor complex assembly, signal propagation efficiency, or transcriptional complex formation downstream of kinase activation^28–30^. Such a mode of action is consistent with the broad inhibitory effects of DAG observed across multiple TLR pathways and transcriptional outputs, and supports the notion that DAG functions as a pathway-level modulator rather than a conventional kinase inhibitor.

## Conclusion

Our findings position DAG as a dual-pathway modulator of TLR signaling, capable of suppressing key inflammatory mediators through inhibition of both NF-κB and IRF3. This dual mechanism provides a strong rationale for further mechanistic and translational studies, with the ultimate goal of evaluating its therapeutic potential in inflammatory and immune-mediated diseases. our findings position DAG as a dual-pathway modulator of TLR signaling, capable of suppressing key inflammatory mediators through inhibition of both NF-κB and IRF3. This dual mechanism provides a strong rationale for further mechanistic and translational studies, with the ultimate goal of evaluating its therapeutic potential in inflammatory and immune-mediated diseases. Such investigations may also reveal whether DAG’s mode of action can be leveraged for multi-target modulation strategies that restore immune balance without complete suppression of innate defense responses.

## Supplementary Information

Below is the link to the electronic supplementary material.

Supplementary Material 1

## Data Availability

The datasets used and/or analysed during the current study are available from the corresponding author on reasonable request.

## Abbreviations

DAG: Dehydroandrographolide
TLR: Toll-like receptor
MyD88: Myeloid differentiation primary response 88
TRIF: TIR-domain-containing adapter-inducing interferon-β
NF-κB: Nuclear factor kappa-light-chain-enhancer of activated B cells
IRF3: Interferon regulatory factor 3
iNOS: Inducible nitric oxide synthase
IFNβ: Interferon beta
IP-10: Interferon gamma-induced protein 10
LPS: Lipopolysaccharide
MALP-2: Macrophage-activating lipopeptide-2
Poly[I:C]: Polyinosinic–polycytidylic acid
DMEM: Dulbecco’s modified Eagle’s medium
DMSO: Dimethyl sulfoxide
SEM: Standard error of the mean
β-gal: β-Galactosidase
