# Supplementary figures and images for: Dehydroandrographolide attenuates Toll-like receptor signaling by dual inhibition of MyD88- and TRIF-dependent pathways
Source: Sci Rep. 2026 Apr 16;16:17658. doi: 10.1038/s41598-026-47514-6 (PMC13243577; doi:10.1038/s41598-026-47514-6)

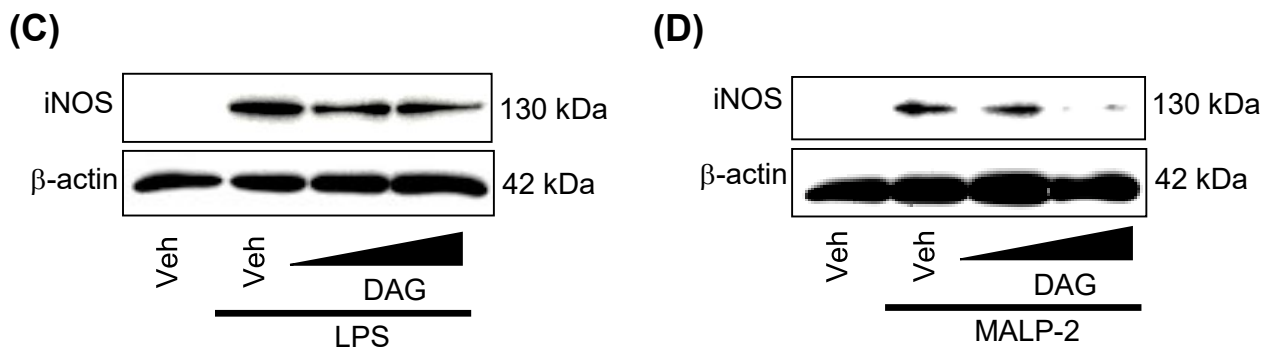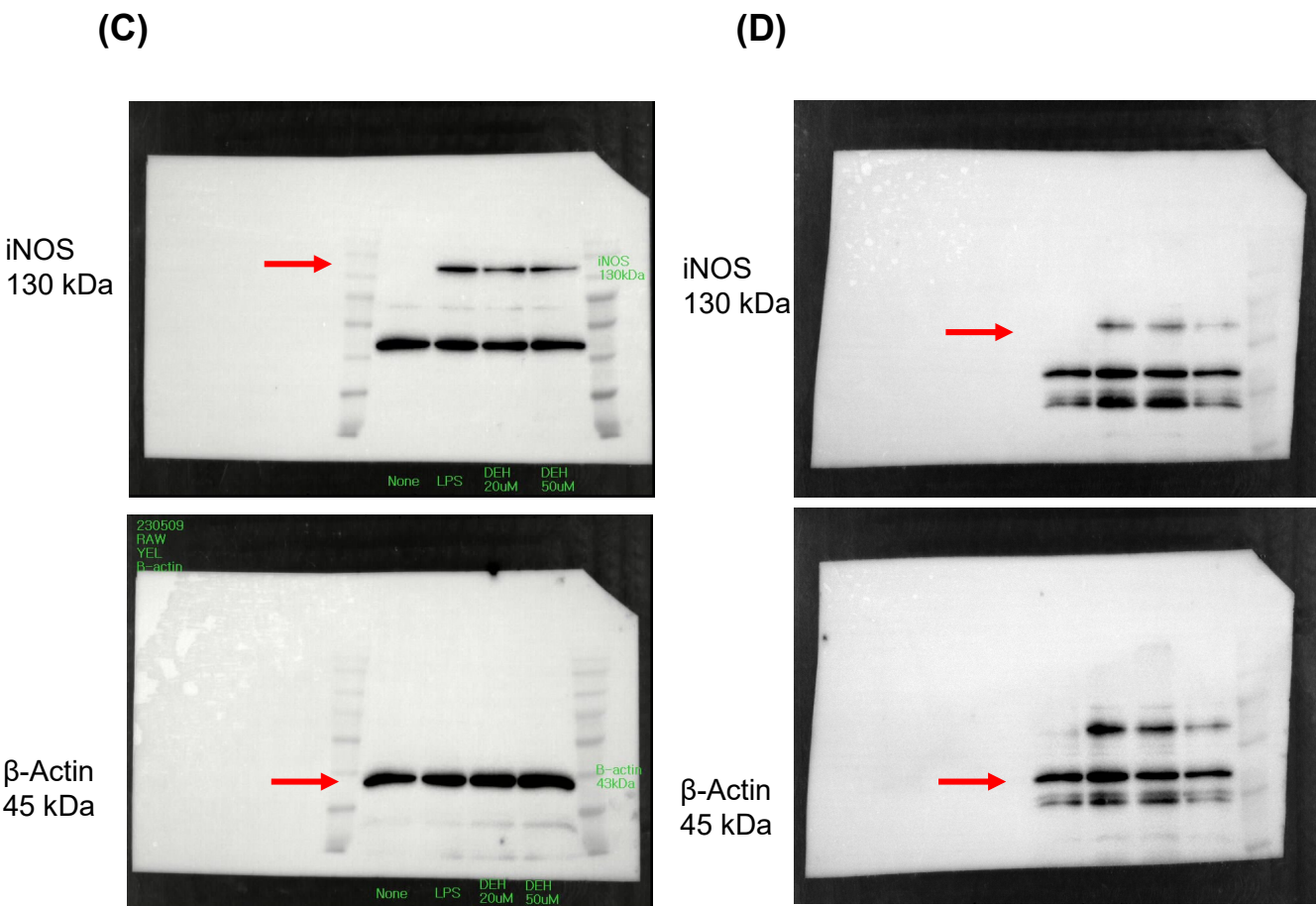

**Fig. 3**

(D)

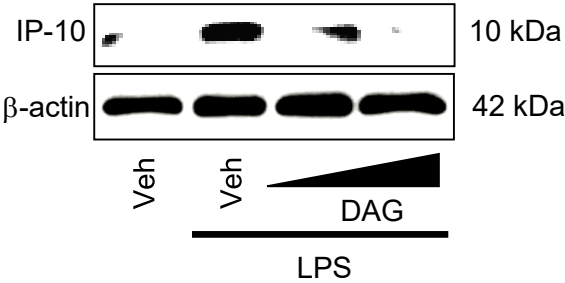

(D)

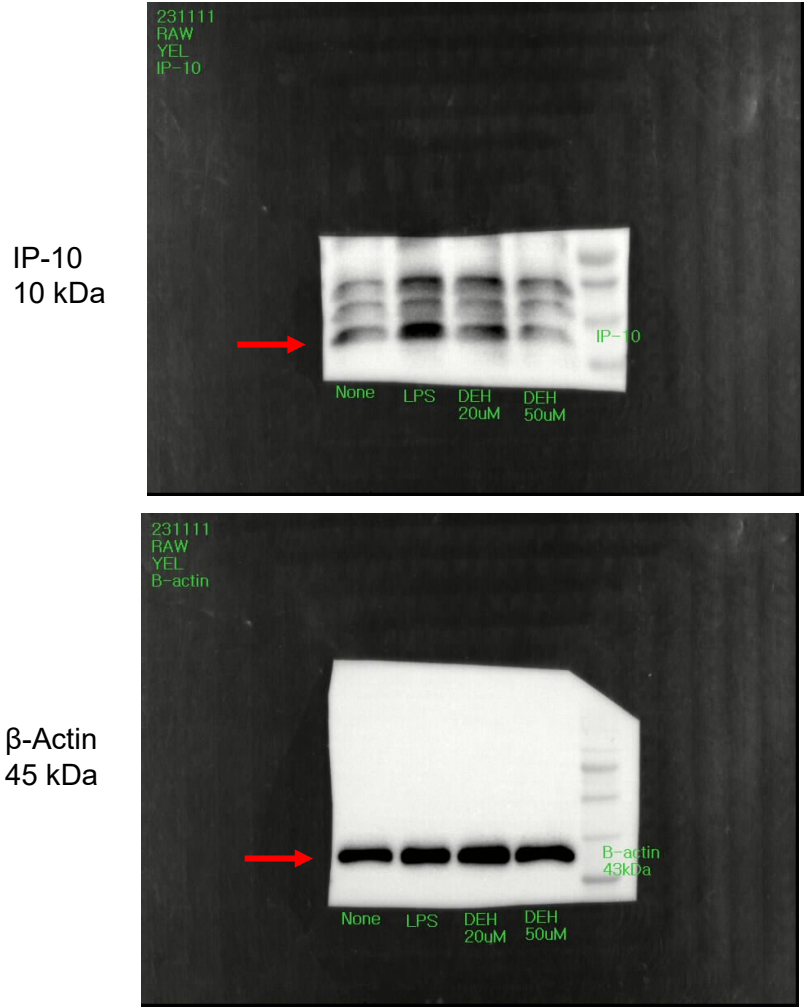

Fig. 5

(C)

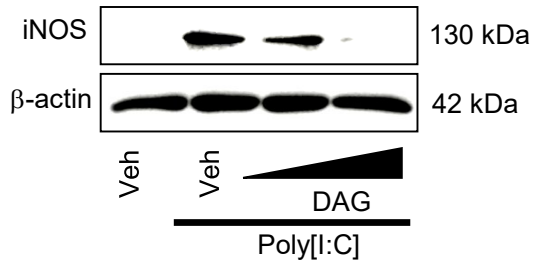

(C)

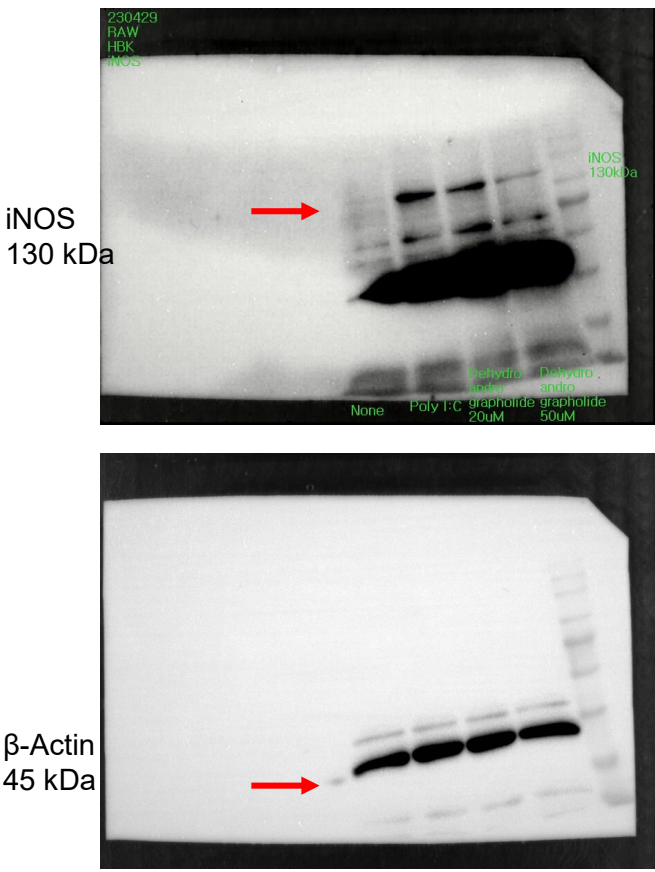

Fig. 6

(D)

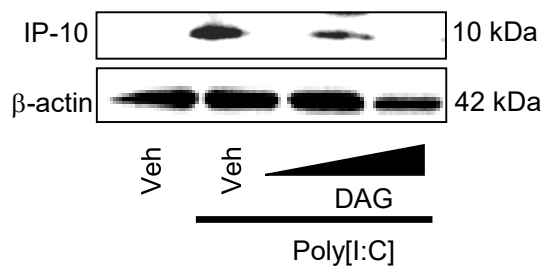

(D)

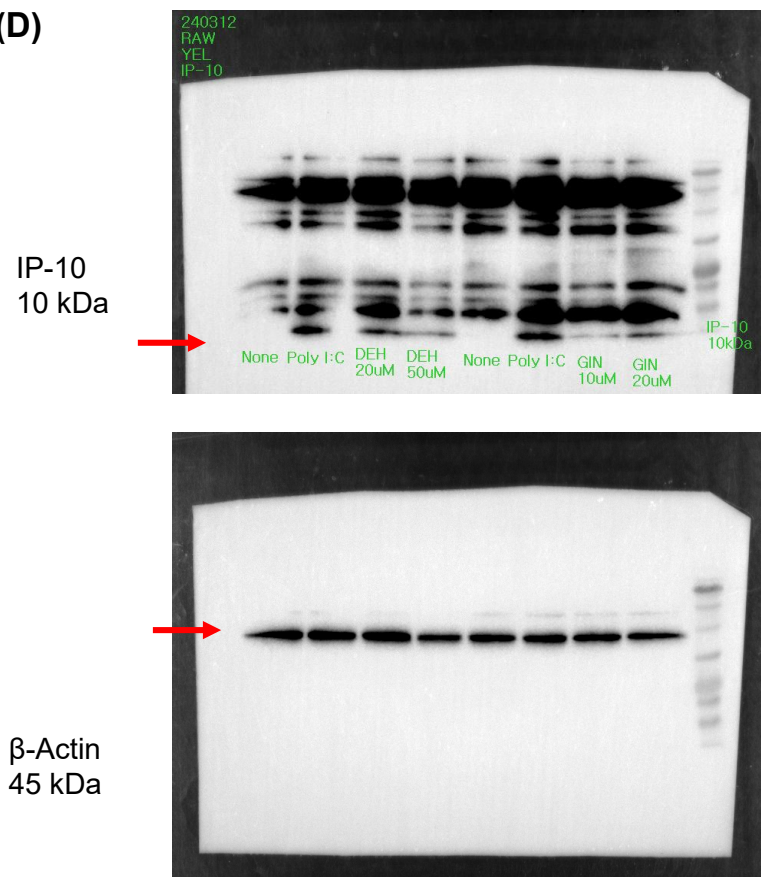

Fig. 7

Supplement: Supplementary file 1 — Supplementary Material 1 [file 41598_2026_47514_MOESM1_ESM.pdf]
